# Mitochondrial Adaptations in Skeletal Muscle Following Incretin‐Based Therapies: In Vitro

**DOI:** 10.1002/jcsm.70254

**Published:** 2026-03-19

**Authors:** Victoria Old, Melanie Davies, Matthew Denniff, Pratik Choudhary, Nicholas Eastley, Robert U. Ashford, Emma Watson

**Affiliations:** ^1^ Division of Cardiovascular Sciences, College of Life Sciences University of Leicester Leicester UK; ^2^ NIHR Leicester Biomedical Research Centre University of Leicester Leicester UK; ^3^ Diabetes Research Centre University of Leicester Leicester UK; ^4^ Division of Respiratory Sciences, College of Life Sciences University of Leicester Leicester UK; ^5^ Leicester Orthopaedics University Hospitals of Leicester Leicester UK; ^6^ Division of Cancer Studies University of Leicester Leicester UK

**Keywords:** GLP‐1RA, mitochondrial health, obesity, skeletal muscle

## Abstract

**Background:**

Incretin‐based therapies such as glucagon‐like peptide‐1 receptor agonists (GLP‐1Ras), dual GLP‐1/GIP agonists and amylin analogues have demonstrated significant weight loss benefits. However, their impact on skeletal muscle mitochondrial function, particularly under metabolic stress, remains unclear. This study aimed to investigate the effects of semaglutide (GLP‐1RA), tirzepatide (dual GLP‐1/GIP agonist) and cagrilintide (amylin analogue) on mitochondrial function in C2C12 skeletal muscle myotubes under both healthy and lipotoxic (palmitic acid‐treated) conditions.

**Methods:**

Differentiated C2C12 myotubes were treated with doses of each drug for 48 h and 5 days. Mitochondrial respiration was assessed using the Seahorse XFp analyser, mitochondrial DNA (mtDNA) copy number and oxidative phosphorylation (OXPHOS) complex protein expression were measured by qPCR and western blotting. Key findings were repeated in primary human skeletal muscle cells.

**Results:**

Palmitic acid (PA) significantly impaired mitochondrial function, reducing basal oxygen consumption rate (OCR) by 22% (*p* = 0.0056) and ATP production by 25% (*p* = 0.0022). In healthy myotubes, semaglutide and cagrilintide transiently reduced basal respiration (↓21%–28%, *p* < 0.05) and ATP production (↓24%–31%, *p* < 0.01) at 48 h, along with reductions in Complexes I, III and IV protein expression, all of which resolved by 5 days. Tirzepatide significantly increased maximal respiration (↑20%–25%, *p* < 0.005) and spare respiratory capacity (↑22%–30%, *p* < 0.005) after 5 days. In PA‐treated myotubes, semaglutide and cagrilintide acutely worsened mitochondrial impairment (↓ATP production by ~20%–25%, *p* < 0.01), but these effects resolved by Day 5. Tirzepatide initially suppressed mitochondrial function (↓ATP production, *p* = 0.0087) but reversed these effects by Day 5, significantly improving ATP production (↑27%–30%, *p* < 0.005), basal respiration (↑20%, *p* = 0.0152), and coupling efficiency. mtDNA content remained unchanged across all conditions. Similar responses were noted in human myotubes, with a transient reduction in respiration for semaglutide and cagrilintide (↓30%–62%, *p* < 0.05) at 48 h and a significant improvement in maximal respiration for tirzepatide at 5 days (↑42%–52%, *p* = 0.0022).

**Conclusion:**

Incretin‐based therapies exert distinct, time and dose‐dependent effects on skeletal muscle mitochondrial function. Tirzepatide promoted sustained improvements in mitochondrial respiration under both healthy and lipotoxic conditions, indicating potential benefits for maintaining skeletal muscle bioenergetic function. These findings underscore the need for further mechanistic studies and suggest that tirzepatide may have the potential to support skeletal muscle health in metabolic disease.

## 1 Introduction

Obesity affects an estimated 880 million adults worldwide as of 2022 [1], with prevalence continuing to rise. Its strong association with comorbidities such as type‐2 diabetes mellitus (T2DM), cardiovascular disease, and cancer underscores the need for effective treatments. Although lifestyle interventions remain fundamental, recent advancements in pharmacotherapy such as glucagon‐like Peptide‐1 Receptor Agonists (GLP‐1Ras) have transformed obesity management. Initially developed for glycaemic control in T2DM, GLP‐1RAs have since been approved for weight loss, showing 10%–15% reductions in total body weight with semaglutide [2, 3] and 15%–20% with dual agonist tirzepatide [4, 5]. Cagrilintide, an amylin analogue, has also demonstrated efficacy [6], and its combination with semaglutide (CagriSema) has shown weight losses of up to 20.4% [7, 8]. These substantial effects have raised concerns over potential loss of skeletal muscle mass (SMM), with studies using GLP‐1RAs reporting up to 40% of weight loss attributable to lean mass [9].

Skeletal muscle is vital for glucose uptake, energy metabolism and physical function. Obesity related mitochondrial dysfunction [10], characterized by reduced biogenesis, impaired oxidative capacity and elevated oxidative stress, can further impair muscle quality [11]. Current evidence on GLP‐1RA's impact on mitochondrial function is limited and inconsistent [12]. Importantly, no studies have assessed the effects of dual agonists or amylin analogues on mitochondrial health in skeletal muscle.

Given the widespread use of these therapies in both diabetic and nondiabetic populations, understanding their impact on skeletal muscle mitochondria is critical. This study aimed to investigate the effects of GLP‐1RAs, dual GLP‐1/GIP agonists and amylin analogues on mitochondrial health in healthy skeletal muscle myotubes and in myotubes exposed to palmitic acid (PA) as a model of obesity‐induced lipotoxic stress.

## 2 Methods

### 2.1 Cell Culture

C2C12 cells used between passages 3–17 were grown in growth medium comprised of Dulbecco's Modified Eagle's Medium (DMEM) (Sigma Aldrich, UK) with 20% foetal bovine serum (FBS) and 1% penicillin streptomycin (PS) (37°C, 5% CO_2_). Myoblasts were differentiated into myotubes using differentiation medium of DMEM containing 2% horse serum and 1% PS once 90% confluency was reached. C2C12 myotubes were used as they are a well‐established and reproducible in vitro model of skeletal muscle that enables controlled mechanistic studies of mitochondrial function. This model provides consistent differentiation and metabolic characteristics, making them an ideal model in which to study the effect of GLP‐1 medication upon mitochondrial function.

Primary human skeletal muscle cells were isolated from skeletal muscle biopsies collected from participants in the ‘Merlin’ Study. Healthy individuals with no significant medical history were recruited from orthopaedic theatre lists during procedures for benign tumour removal, and muscle biopsies were obtained via the open biopsy technique. The study received ethical approval from the UK National Research Ethics Committee (Ref: 15/EM/0467), and all participants provided written informed consent. The trial was conducted in accordance with the Declaration of Helsinki. Cell isolation was performed as previously described [13]. Key experiments were replicated in primary human skeletal muscle cells to confirm the main findings observed in C2C12 myotubes.

### 2.2 Cell Treatments

Prior optimization of drug and Palmitic Acid (PA) concentrations was performed using the Alamar Blue viability assay and normalized to total protein concentration (Supporting Information). Two skeletal muscle cells were employed in the C2C12 cell line: (1) healthy myotubes and (2) PA‐treated myotubes, which were subjected to a lipotoxic challenge mimicking metabolic dysfunction commonly observed in obesity [14].

Cells were treated in serum‐free media (DMEM with 1% PS), with drugs applied for either 48 h or 5 days (media changed every 2 days). The following concentrations were used: Semaglutide: 1 nM (sema‐low) and 400 nM (sema‐high), tirzepatide: 10 nM (tirze‐low) and 400 nM (tirze‐high), cagrilinitide: 10 nM (cagril‐low) and 400 nM (cagril‐high). The low‐dose ranges (1–10 nM) were selected to reflect systemic concentrations achievable in clinical and preclinical models [15, 16, 17], particularly with standard therapeutic dosing of GLP‐1RA and amylin analogues. The high dose (400 nM) was included as a supraphysiological condition to explore potential receptor saturation effects and signalling responses not typically observed in vivo but relevant for mechanistic insight.

For PA‐treated myotubes, 0.2 mM PA was applied to serum‐free media and incubated with cells for 24 h to induce lipotoxic stress. Following PA exposure, cells were washed with PBS before application of drug treatment.

### 2.3 Mitochondrial Respiration Assay

Mitochondrial respiration was analysed using the Mito Stress Test by XFp Analyser (Seahorse Bioscience, Agilent Technologies, USA). Cells were plated at a density of 20 000 cells/well in Xfe24 well plates. Differentiation media was changed every 48 h until myotubes formed. Once formed, cells were treated with the incretin drugs as described above. Once incubation times were completed, the media were changed to Xf assay media consisting of 2‐mM L‐glutamine, 1‐mM pyruvate and 10‐mM glucose (0.5 mL per well) and incubated in a non‐CO_2_ incubator for 45–60 min prior to assay run. Stock solutions of Oligomycin (2.5 uM), FCCP (1 uM), rotenone and antimycin (0.5 uM/0.5 uM) were prepared in Xf assay media and added to the cartridge. The assay was run with the following cycles: Basal, 3 cycles: 3 min mix, 0 min wait, 3 min measure; inject Port A (oligomycin) 3 cycles: 3 min mix, 0 min wait, 3 min measure; inject Port B (FCCP) 3 cycles: 3 min mix, 0 min wait, 3 min measure; inject Port C (rotenone/antimycin) 3 cycles: 3 min mix, 0 min wait, 3 min measure. Cells were lysed using RIPA buffer post assay to measure protein content using the Bio‐Rad DC protein assay (Bio‐Rad, CA, USA).

### 2.4 Mt. DNA Copy Number

Cells were lysed in trizol for DNA extraction and quantified using the nanodrop (Geneflow Ltd., Litchfield, UK). Quantitative Polymerase Chain Reaction (qPcr) was performed using 0.5 μg of DNA with TaqMan probes for ND1 (Mm04225274_s1) and Actb (Mm02619580_g1). Absolute mtDNA copy number was determined by comparing ND1 amplification to a standard curve generated from a sample with known mtDNA content. Final values were normalized by ND1/ACTB to account for nuclear DNA content per cell.

### 2.5 Western Blot

C2C12 cells were grown and treated as described above. Cells were lysed with RIPA buffer and protein concentration determined using the Bio‐Rad protein assay (Bio‐Rad, CA, USA). Lysates containing 30 ug of protein were run on SDS‐PAGE. Proteins were transferred onto PDVF membranes (GE Healthcare, Germany) and blocked for 1 h. Membranes were incubated with the primary antibody for 36 h: GLP‐1R was used at 1:500 (Santa Cruz Biotechnology, TX, USA), OXPHOS antibody cocktail (Invitrogen, MA, USA) was used at 1:1500. Band intensity was captured using a Bio‐Rad ChemiDoc touch instrument and quantified using Image Lab Software (Bio‐Rad, CA, USA). Total protein visualized by stain‐free imaging using ChemiDoc touch instrument (Bio‐Rad, UK) was used as the loading control for normalization of protein expression in western blot analysis.

### 2.6 Statistical Analysis

All data are presented as mean ± SD Normality was tested using the Shapiro–Wilk test. When comparing within time points, a one‐way ANOVA was performed with Tukey's post hoc or a Kruskal–Wallis test with Dunn's post hoc was performed as appropriate. Statistical analysis was performed using GraphPad Prism 10.2.3. Statistical significance was accepted as *p* < 0.05.

## 3 Results

### 3.1 Mitochondrial Respiration

#### 3.1.1 Healthy Semaglutide

**FIGURE 1:**
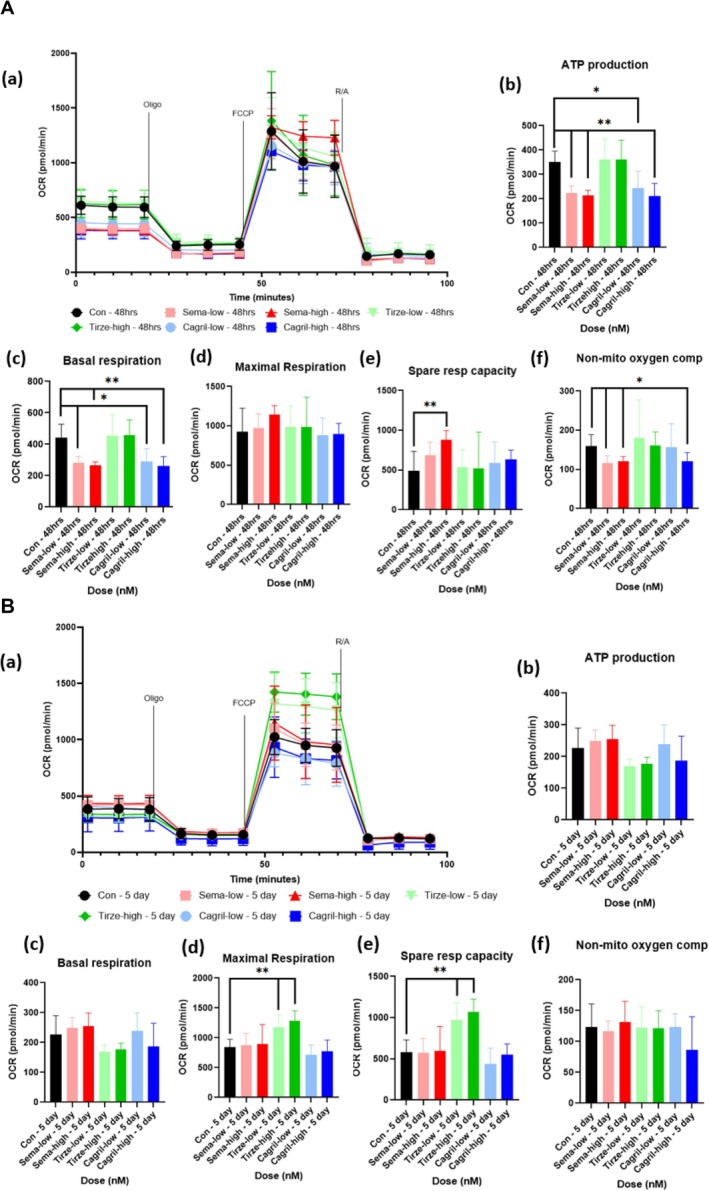
(A) Seahorse mito stress test results of healthy C2C12 myotubes dosed with semaglutide, tirzepatide and cagrilintide for 48 h. (a) OCR graph following sequential administration of oligomycin, FCCP, rotenone and antimycin‐a. (b–f) Quantification of: (b) ATP production, (c) basal respiration, (d) maximal respiration, (e) spare respiratory capacity and (f) non‐mito oxygen consumption. Data are presented as mean ± SD (*n* = 6 per group). Statistical analysis was performed using one‐way ANOVA with Tukey's post hoc test or Kruskal–Wallis test with Dunn's post hoc test for nonparametric data. For ATP production (b): cagril‐low vs. control, *p* < 0.05 (*), sema‐low vs control, sema‐high vs control, cagril‐high vs control, *p* < 0.005 (**); basal respiration (c): sema‐low vs control, cagril‐low vs control, *p* < 0.05, sema‐high vs control, cagril‐high vs control, *p* < 0.005; spare respiratory capacity (e): sema‐high vs control, *p* < 0.005; nonmitochondrial oxygen consumption (f): sema‐low vs control, sema‐high vs control, cagril‐high vs control, *p* < 0.05. Abbreviations: OCR = oxygen consumption rate, oligo = oligomycin, R/A = rotenone and antimycin‐a. (B) Seahorse mito stress test results of healthy C2C12 myotubes dosed with semaglutide, tirzepatide and cagrilintide for 5 days. (a) OCR graph following sequential administration of oligomycin, FCCP, rotenone and antimycin‐a. (b–f) Quantification of: (b) ATP production, (c) basal respiration, (d) maximal respiration, (e) spare respiratory capacity and (f) non‐mito oxygen consumption. Statistical analysis was performed using one‐way ANOVA with Tukey's post hoc test or Kruskal–Wallis test with Dunn's post hoc test for nonparametric data. For maximal respiration (d): tirze‐low vs. control, tirze‐high vs. control, *p* < 0.005 (**); spare respiratory capacity (e): tirze‐low vs. control, tirze‐high vs. control, *p* < 0.005. Abbreviations: OCR = oxygen consumption rate, oligo = oligomycin, R/A = rotenone and antimycin‐a.

**FIGURE 2:**
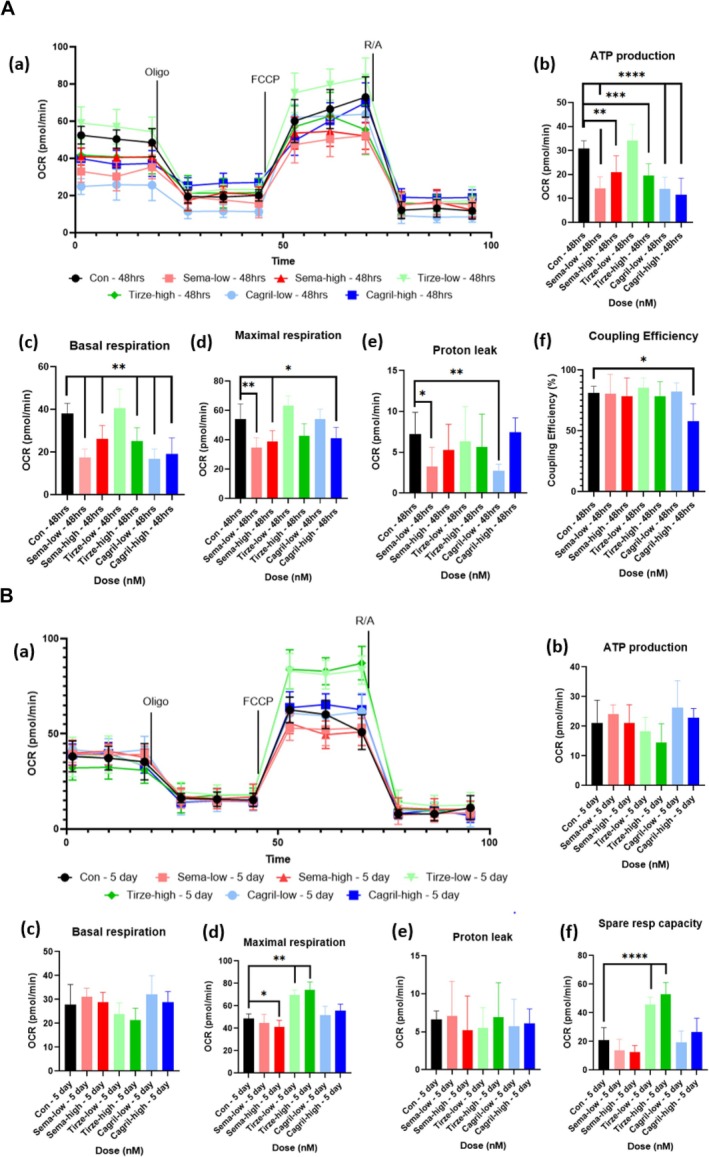
(A) Seahorse mito stress test results of healthy primary human myotubes dosed with semaglutide, tirzepatide and cagrilintide for 48 h. (a) OCR graph following sequential administration of oligomycin, FCCP, rotenone and antimycin‐a. (b–f) Quantification of: (b) ATP production, (c) basal respiration, (d) maximal respiration, ATP production, (e) proton leak and (f) coupling efficiency. Data are presented as mean ± SD (*n* = 6 per group). Statistical analysis was performed using one‐way ANOVA with Tukey's post hoc test or Kruskal–Wallis test with Dunn's post hoc test for nonparametric data. For basal respiration (b): sema‐low vs. control, *p* < 0.005 (**), sema‐high vs. control, *p* < 0.005, tirze‐high vs. control, *p* < 0.005, cagril‐low vs. control, *p* < 0.005,uction, (c) basal respiration, (d) maximal respiration, ATP production, (e) proton leak and (f) coupling efficiency. Data are presented as mean ± SD (*n* = 6 per group). Statistical analysis was performed using one‐way ANOVA with Tukey's post hoc test or Kruskal–Wallis test with Dunn's post hoc test for nonparametric data. For basal respiration (b): sema‐low vs. control, *p* < 0.005 (**), sema‐high vs. control, *p* < 0.005, tirze‐high vs. control, *p* < 0.005, cagril‐low vs. control, *p* < 0.005, cagril‐high vs. control, *p* < 0.005; maximal respiration (c): sema‐low vs. control, *p* < 0.005, sema‐high vs. control, *p* < 0.05 (*), cagril‐high vs. control, *p* < 0.05; ATP production (d): sema‐low vs. control, *p* < 0.0001 (****), sema‐high vs. control, *p* < 0.005, tirze‐high vs. control, *p* < 0.0001 (***), cagril‐low vs. control, *p* < 0.0001, cagril‐high vs. control, *p* < 0.0001; proton leak (e): sema‐low vs. control, *p* < 0.05, cagril‐low vs. control, *p* < 0.005; coupling efficiency (f): cagril‐low vs. control, *p* < 0.05. Abbreviations: OCR = oxygen consumption rate, oligo = oligomycin, R/A = rotenone and antimycin‐a. (B) Seahorse mito stress test results of healthy primary human myotubes dosed with semaglutide, tirzepatide and cagrilintide for 5 days. (a) OCR graph following sequential administration of oligomycin, FCCP, rotenone and antimycin‐a. (b–f) Quantification of: (b) ATP production, (c) basal respiration, (d) maximal respiration, (e) proton leak and (f) spare respiratory capacity. Data are presented as mean ± SD (*n* = 6 per group). Statistical analysis was performed using one‐way ANOVA with Tukey's post hoc test or Kruskal–Wallis test with Dunn's post hoc test for nonparametric data. For maximal respiration (d): sema‐high vs. control, *p* < 0.05 (*), tirze‐low vs. control, *p* < 0.005 (**), tirze‐high vs. control, *p* < 0.005; spare respiration capacity (f): tirze‐low vs. control, *p* < 0.0001 (****), tirze‐high vs. control, *p* < 0.0001. Abbreviations: OCR = oxygen consumption rate, oligo = oligomycin, R/A = rotenone and antimycin‐a.

In healthy C2C12 myotubes, Semaglutide treatment induced several transient changes in mitochondrial respiration at 48 h (Figure 1A) that were not sustained at the 5‐day time point (Figure 1B). At 48 h, both sema‐low and sema‐high significantly reduced ATP‐linked respiration compared to the healthy control (*p* = 0.002). Basal respiration was significantly reduced after 48 h following both sema‐low (*p* = 0.014) and sema‐high (*p* = 0.003) compared to the healthy control. Sema‐high significantly increased spare respiratory capacity at 48 h (*p* = 0.0087) suggesting an acute enhancement in mitochondrial flexibility. Nonmitochondrial oxygen consumption was significantly reduced by both doses at 48 h (*p* < 0.05). Despite reductions in ATP‐linked and basal respiration at 48 h, coupling efficiency (calculated by dividing ATP‐linked respiration (basal respiration—oligomycin‐inhibited respiration) by basal respiration, expressed as a percentage) remained unchanged across all conditions (*p* > 0.05), indicating that the proportion of oxygen consumption used for ATP production relative to total basal respiration was preserved. Consistent with these findings, semaglutide treatment in primary human myotubes produced a similar pattern, with reductions in basal respiration (*p* = 0.0022) and ATP production at 48 h for both sema‐low (*p* < 0.0001) and sema‐high (*p* = 0.0094) conditions (Figure 2A). In addition, there was a suppression in maximal respiration at 48 h for both sema‐low (*p* = 0.0029) and sema‐high (*p* = 0.0135); this reduction continued at the 5‐day point for sema‐high (*p* = 0.0411) (Figure 2B). Proton leak was also significantly altered after 48 h in the sema‐low group (*p* = 0.026). Overall, semaglutide induced a transient suppression of mitochondrial respiration, characterized by reduced basal and ATP‐linked respiration that resolved with prolonged exposure. These results in both C2C12 and primary human myotubes indicate semaglutide's effects on mitochondrial function are largely conserved between murine and human, with subtle differences in dose and time‐dependent sensitivity.

#### 3.1.2 Healthy Tirzepatide

In healthy C2C12 myotubes, tirzepatide treatment had minimal impact on mitochondrial function at 48 h (Figure 1A) but elicited notable enhancements after 5 days (Figure 1B). Neither tirze‐low or tirze‐high significantly changed maximal respiration after 48 h (*p* > 0.05); however, 5 days of incubation led to a significant increase in maximal respiration with both tirze‐low (*p* = 0.0043) and tirze‐high (*p* = 0.0022). This was accompanied by a significant elevation in spare respiratory capacity after 5 days in both doses (*p* = 0.0022), indicating an enhanced ability of mitochondria to respond to increased energy demand. Despite increases in maximal respiration and spare respiratory capacity over time, coupling efficiency remained unchanged throughout (*p* > 0.05), suggesting that the proportion of oxygen consumption dedicated to ATP synthesis relative to basal respiration was maintained. In primary human myotubes, tirzepatide exhibited a comparable pattern, with both doses significantly increasing maximal respiration (*p* = 0.0022) and spare respiratory capacity (*p* < 0.0001) by 5 days (Figure 2B), mirroring the enhancements observed in C2C12 myotubes. Further to this, in tirze‐high, there was a reduction at 48 h in basal respiration (*p* = 0.0022) and ATP production (*p* = 0.0008) (Figure 2A). Together, the data suggest that tirzepatide selectively promotes mitochondrial adaptability in healthy myotubes over long durations.

#### 3.1.3 Healthy Cagrilintide

In healthy C2C12 myotubes, both doses of cagrilintide induced temporary reductions in aspects of mitochondrial respiration at 48 h (Figure 1A) that resolved by the 5‐day time point (Figure 1B), following a similar pattern to semaglutide. At 48 h, there were significant reductions in ATP‐linked respiration for cagril‐low (*p* = 0.02) and cagril‐high (*p* = 0.002) along with reductions in basal respiration for cagril‐low (*p* = 0.024) and cagril‐high (*p* = 0.002) compared to the healthy controls, indicating a short‐term suppression of mitochondrial activity. Nonmitochondrial oxygen consumption was significantly decreased at 48 h in cagril‐high (*p* = 0.026). Despite reductions in basal and ATP‐linked respiration at 48 h, coupling efficiency remained unchanged at both time points (*p* > 0.05), indicating that the proportion of oxygen consumption supporting ATP synthesis relative to basal respiration was preserved. Similarly, in primary human myotubes, both doses lead to significant reductions in ATP production (*p* < 0.0001) and basal respiration (*p* = 0.0022) at 48 h (Figure 2A). Additionally, there was a reduction at 48 h in maximal respiration for cagril‐high (*p* = 0.0292) and in proton leak for cagril‐low (*p* = 0.0043). Spare respiratory capacity was increased at 48 h in cagril‐low (*p* = 0.0046), suggesting a compensatory enhancement in mitochondrial reserve. Coupling efficiency was significantly reduced at 48 h in cagril‐high (*p* = 0.0231), indicating a potential short‐term decrease in mitochondrial efficiency. Overall, these findings demonstrate that cagrilintide exerts transient, dose‐dependent effects on mitochondrial respiration in human myotubes, largely paralleling the temporal pattern observed in murine cells, with all effects resolving by 5 days.

### 3.2 PA Treatment‐Induced Mitochondrial Dysfunction

Comparing healthy and PA‐treated myotubes revealed a selective impairment of basal and ATP‐linked mitochondrial respiration under lipotoxic conditions. PA treatment significantly reduced basal OCR (*p* = 0.0056) and ATP‐linked respiration (*p* = 0.0022), indicating diminished mitochondrial efficiency in response to lipid‐induced stress.

#### 3.2.1 PA‐Treated Semaglutide

**FIGURE 3:**
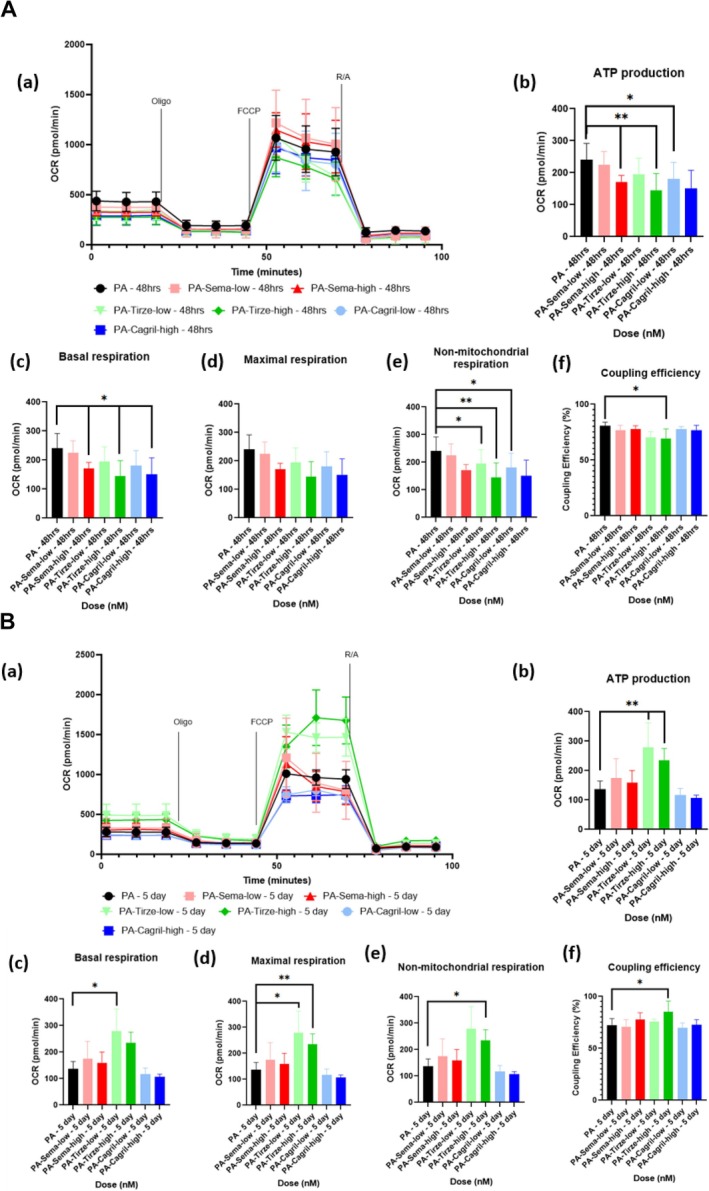
(A) Seahorse mito stress test results of PA‐dosed C2C12 myotubes dosed with semaglutide, tirzepatide and cagrilintide for 48 h. (a) OCR graph following sequential administration of oligomycin, FCCP, rotenone and antimycin‐a. (b–f) Quantification of: (b) ATP production, (c) basal respiration, (d) maximal respiration, (e) nonmitochondrial respiration and (f) coupling efficiency. Data are presented as mean ± SD (*n* = 6 per group). Statistical analysis was performed using one‐way ANOVA with Tukey's post hoc test or Kruskal–Wallis test with Dunn's post hoc test for nonparametric data. For ATP production (b): sema‐high vs. control, tirze‐high vs. control, *p* < 0.005 (**), cagril‐low vs. control, *p* < 0.05 (*); basal respiration (c): sema‐high vs. control, tirze‐high vs. control and cagril‐high vs. control, *p* < 0.05; nonmitochondrial respiration (e): tirze‐low vs. control, cagril‐low vs. control, *p* < 0.05, tirze‐high vs. control, *p* < 0.005; coupling efficiency (f): tirze‐high vs. control, *p* < 0.05. Abbreviations: OCR = oxygen consumption rate, oligo = oligomycin, R/A = rotenone and antimycin‐a. (B) Seahorse mito stress test results of PA‐dosed C2C12 myotubes dosed with semaglutide, tirzepatide and cagrilintide for 5 days. (a) OCR graph following sequential administration of oligomycin, FCCP, rotenone and antimycin‐a. (b–f) Quantification of (b) ATP production, (c) basal respiration, (d) maximal respiration, (e) nonmitochondrial oxygen consumption and (f) coupling efficiency. Statistical analysis was performed using one‐way ANOVA with Tukey's post hoc test or Kruskal–Wallis test with Dunn's post hoc test for nonparametric data. For ATP production (b): tirze‐low vs. control, tirze‐high vs. control, *p* < 0.005 (**); basal respiration (c): tirze‐low vs. control, *p* < 0.05 (*); maximal respiration (d): tirze‐low vs. control, *p* < 0.05, tirze‐high vs. control, *p* < 0.005; nonmitochondrial respiration (e): tirze‐high vs. control, *p* < 0.05; coupling efficiency (f): tirze‐high vs. control, *p* < 0.05. Abbreviations: OCR = oxygen consumption rate.

In PA‐treated C2C12 myotubes, high‐dose semaglutide further reduced aspects of mitochondrial function at 48 h (Figure 3A), but these effects normalized after 5 days (Figure 3B). Specifically, there was a significant decrease in ATP production (*p* = 0.0087) and basal respiration (*p* = 0.0152) in the sema‐high group at 48 h compared to PA‐treated myotubes suggesting an acute exacerbation of the lipotoxic phenotype. Coupling efficiency remained unchanged in all treatment conditions (*p* > 0.05), indicating that despite transient reductions in respiratory output, the proportion of oxygen consumption supporting ATP production relative to basal respiration was not compromised. These findings suggest that high‐dose semaglutide may initially impair mitochondrial function in PA‐treated myotubes, though this effect appears to resolve with prolonged exposure.

#### 3.2.2 PA‐Treated Tirzepatide

In PA‐treated C2C12 myotubes, tirzepatide had distinct temporal effects on mitochondrial function. At 48 h (Figure 3A), ATP production was significantly decreased in the PA‐tirze‐high compared to PA‐treated control (*p* = 0.0087), and basal respiration was also significantly reduced (*p* = 0.0411), suggesting an initial suppression of mitochondrial output. After 5 days (Figure 3B) ATP production was significantly increased in both PA‐tirze‐low (*p* = 0.0043) and high‐dose groups (*p* = 0.0043), and basal respiration was significantly elevated in the low‐dose group (*p* = 0.0152), indicating a delayed enhancement of mitochondrial function. Maximal respiration showed a significant increase at the 5‐day mark in both PA‐tirze‐low (*p* = 0.0142) and PA‐tirze‐high (*p* = 0.0073) compared to the PA‐treated control, indicating enhanced respiratory capacity under prolonged treatment. Interestingly, nonmitochondrial oxygen consumption was significantly reduced in both PA‐tirze‐low (*p* = 0.0411) and PA‐tirze‐high (*p* = 0.0087) groups at 48 h, but significantly increased at 5 days with the high dose (*p* = 0.0043), suggesting potential off‐target or compensatory effects over time. Coupling efficiency was significantly decreased at 48 h in the high‐dose group (*p* = 0.041), consistent with reduced ATP production relative to basal oxygen consumption, but showed a significant increase at 5 days (*p* = 0.02) indicating an enhanced proportion of respiration devoted to ATP production following prolonged treatment. These findings indicate that tirzepatide exerts biphasic effects on mitochondrial respiration under lipotoxic conditions, with early suppression, that was not observed in non‐PA treatment myotubes, followed by a recovery or enhancement with prolonged treatment.

#### 3.2.3 PA‐Treated Cagrilintide

In PA‐treated C2C12 myotubes, high‐dose cagrilintide treatment led to a significant reduction in ATP production (*p* = 0.026) and basal respiration (*p* = 0.0411) at 48 h compared with PA‐treated controls, indicating a transient impairment of mitochondrial function under metabolic stress. These effects were no longer evident at 5 days (Figure 3B) (*p* > 0.05), confirming their short‐lived response. Additionally, nonmitochondrial oxygen consumption was significantly decreased in the low‐dose group at 48 h (*p* = 0.0411). Coupling efficiency remained unchanged at both time points and doses (*p* > 0.05), indicating that although mitochondrial respiration was acutely suppressed, the proportion of oxygen consumption dedicated to ATP production was not altered. These findings suggest that cagrilintide may temporarily exacerbate mitochondrial inefficiency and reduce nonmitochondrial oxygen use in PA‐treated myotubes but does not produce lasting respiratory impairments with prolonged exposure.

### 3.3 Mitochondrial Copy Number

Analysis of mitochondrial (mtDNA) copy number revealed no significant differences in mtDNA copy number between any of the drug treatment groups and the control at either the 48 h or 5‐day time point in both the healthy and PA‐treated myotubes (*p* > 0.05, Supporting Information). Regardless of drug dose, mtDNA copy number remained stable, suggesting that the treatments did not alter mitochondrial biogenesis or degradation within the time frame. This uniform lack of an effect across all compounds indicates that mtDNA copy number responds similarly to each treatment, consistent with a shared class‐level effect rather than compound‐specific differences.

### 3.4 Mitochondrial Complex Protein Abundance

#### 3.4.1 Healthy Semaglutide

**FIGURE 4:**
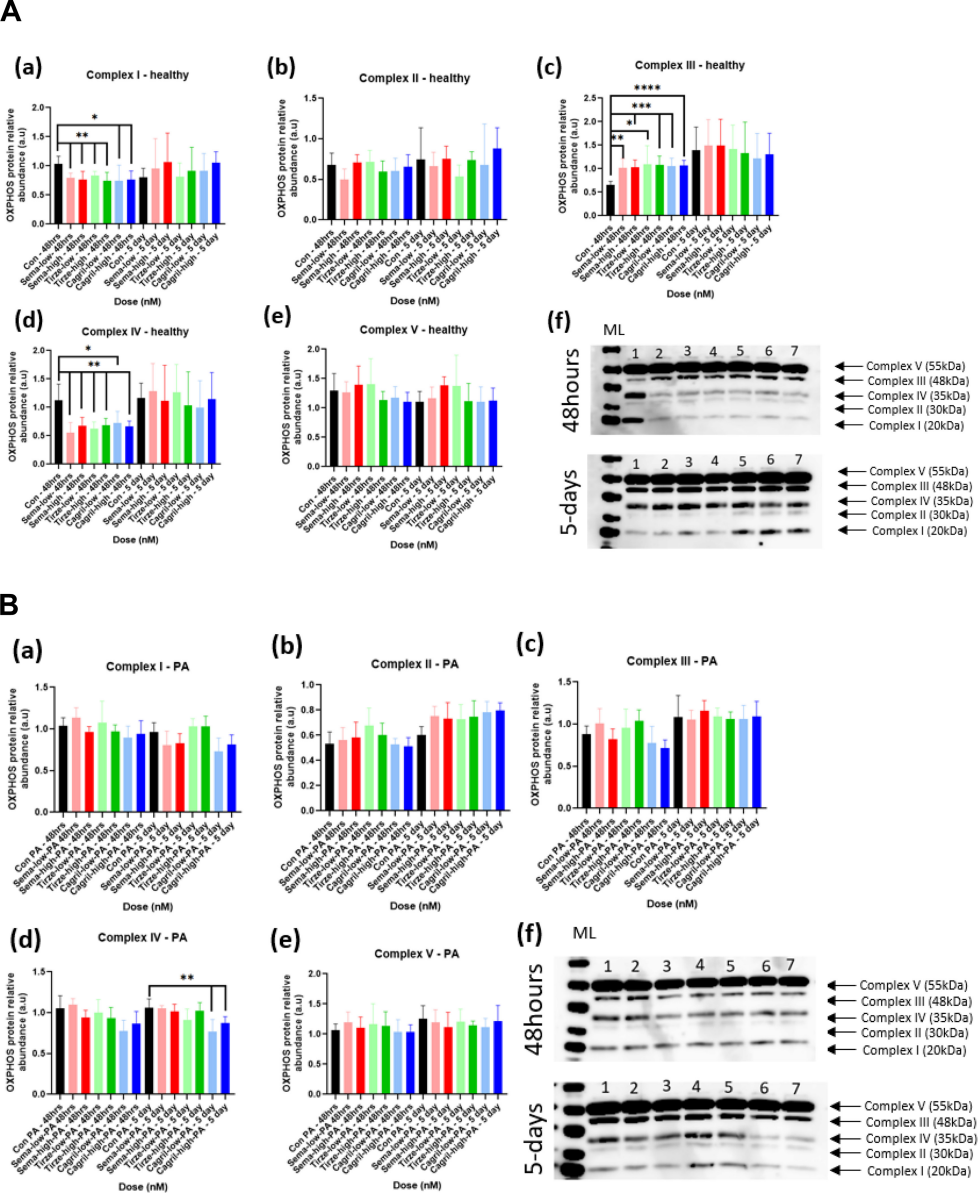
(A) OXPHOS protein expression results of healthy C2C12 myotubes following high and low semaglutide, tirzepatide and cagrilintide dosing over 48 h and 5 days. Quantification of (a–e): (a) OXPHOS Complex I, (b) OXPHOS Complex II, (c) OXPHOS Complex III, (d) OXPHOS Complex IV, (e) OXPHOS Complex V. (f) Representative western blot of OXPHOS complexes after 48 h and 5 days incubation with: (1) control, (2) sema‐low, (3) sema‐high, (4) tirze‐low, (5) tirze‐high, (6) cagril‐low and (7) cagril‐high. Data are presented as mean ± SD (*n* = 6 per group). Statistical analysis was performed using one‐way ANOVA with Tukey's post hoc test or Kruskal–Wallis test with Dunn's post hoc test for nonparametric data. For Complex I (a): sema‐low 48 h vs. control 48 h, sema‐high 48 h vs. control 48 h, tirze‐low 48 h vs. control 48 h, tirze‐high 48 h vs. control 48 h, *p* < 0.005 (**), cagril‐low 48 h vs. control 48 h, cagril‐high 48 h vs. control 48 h, *p* < 0.05 (*); Complex III (c): sema‐low 48 h vs. control 48 h, *p* < 0.005, tirze‐low 48 h vs. control 48 h, *p* < 0.05, sema‐high 48 h vs. control 48 h, tirze‐high 48 h vs. control 48 h, cagril‐low 48 h vs. control 48 h, *p* < 0.0001(***), cagril‐high 48 h vs. control 48 h, *p* < 0.00001 (****); Complex IV (d): sema‐low 48 h vs. control 48 h, sema‐high 48 h vs. control 48 h, tirze‐low 48 h vs. control 48 h, tirze‐high 48 h vs. control 48 h, cagril‐high 48 h vs. control 48 h, *p* < 0.005, cagril‐low 48 h vs. control 48 h, *p* < 0.05. Abbreviations: a.u = arbitrary units, ML = molecular weight ladder. (B) OXPHOS protein expression results of PA‐conditioned C2C12 myotubes following high and low doses of semaglutide, tirzepatide and cagrilintide dosing over 48 h and 5 days. Quantification of (a–e): (a) OXPHOS Complex I, (b) OXPHOS Complex II, (c) OXPHOS Complex III, (d) OXPHOS Complex IV and (e) OXPHOS Complex V. (f) Representative western blot of OXPHOS complexes after 48‐h and 5‐day incubation with: (1) control, (2) sema‐low, (3) sema‐high, (4) tirze‐low, (5) tirze‐high, (6) cagril‐low and (7) cagril‐high. Data are presented as mean ± SD (*n* = 6 per group). Statistical analysis was performed using one‐way ANOVA with Tukey's post hoc test or Kruskal–Wallis test with Dunn's post hoc test for nonparametric data. For Complex IV (d): cagril‐low 5 days vs. control 5 days, cagril‐high 5 days vs. control 5 days, *p* < 0.005 (**). Abbreviations: a.u = arbitrary units, ML = molecular weight ladder.

Western blot analysis of oxidative phosphorylation (OXPHOS) complex subunit expression revealed a selective, transient modulation of mitochondrial respiratory chain components in semaglutide‐treated healthy C2C12 myotubes (Figure 4A). At 48 h, both low and high doses of Semaglutide resulted in a significant reduction in Complex I (sema‐low: *p* = 0.0022, sema‐high: *p* = 0.0087) and Complex IV (sema‐low: *p* = 0.0022, sema‐high: *p* = 0.0022) protein levels compared to healthy control myotubes, indicating differential regulation of specific OXPHOS complexes, while Complex III expression was significantly increased (sema‐low: *p* = 0.0024, sema‐high: *p* = 0.0004). This pattern indicates a transient and differential regulation of OXPHOS complexes, with semaglutide suppressing Complexes I and IV, but enhancing Complex III expression, consistent with the overall pattern seen across the other compounds.

#### 3.4.2 Healthy Tirzepatide

In healthy C2C12 myotubes, tirzepatide treatment resulted in a transient, dose‐independent alteration in OXPHOS protein expression (Figure 4A). At 48 h, both low and high doses of tirzepatide led to a significant decrease in Complex I (*p* < 0.0087) and Complex IV protein levels compared to healthy controls (*p* < 0.0022), indicating a potential early suppression of specific mitochondrial respiratory components. In contrast, a significant increase in Complex III expression (tirze‐low: *p* = 0.0229, tirze‐high: *p* = 0.0005) was observed at the same time point for both doses, suggesting a compensatory or targeted upregulation of this complex. This indicates that tirzepatide's effects on OXPHOS protein expression are selective and time‐limited.

#### 3.4.3 Healthy Cagrilintide

After 48 h, both low and high doses of cagrilintide resulted in a significant reduction in the expression of Complex I (cagril‐low: *p* = 0.0415, cagril‐high: *p* = 0.0152) and Complex IV proteins (cagril‐low: *p* = 0.0152, cagril‐high: *p* = 0.0022) compared to the healthy control group, indicating a downregulation of these specific components of the mitochondrial respiratory chain (Figure 4A). Conversely, a significant increase in Complex III expression was observed at the same time point for both doses (cagril‐low: *p* = 0.0003, cagril‐high: *p* < 0.0001), suggesting a selective upregulation of this complex, which may lead to inefficient ATP production or increased ROS production. These findings suggest that cagrilintide selectively modulates specific OXPHOS complexes at 48 h but does not exert lasting alterations in mitochondrial respiratory chain protein expression under the time frame.

Taken together, these findings suggest that, like mtDNA copy number, a class‐related pattern is present across all three compounds, characterized by a transient downregulation of Complexes I and IV alongside upregulation of Complex III, indicating a shared short‐term modulation of mitochondrial respiratory chain components by incretin‐based therapies.

#### 3.4.4 PA‐Treated Semaglutide and Tirzepatide

At 48 h and 5‐days (Figure 4B), there were no significant differences in the expression levels of any of the OXPHOS Complexes (I–V) between the semaglutide + PA‐treated groups and the PA‐treated control (*p* > 0.05). These results suggest that semaglutide and tirzepatide, when coadministered with palmitic acid, do not significantly alter the expression of mitochondrial OXPHOS complexes over 5 days.

#### 3.4.5 PA‐Treated Cagrilintide

At 48 h (Figure 4B), no significant differences in the expression of any OXPHOS complexes were observed (*p* > 0.05). At 5 days, a significant decrease in Complex IV expression was detected in both the low‐ and high‐dose cagrilintide + PA groups compared to the PA‐treated control (cagril‐low: *p* = 0.0043, cagril‐high: *p* = 0.026). These findings suggest that cagrilintide, when coadministered with palmitic acid, selectively reduces Complex IV expression at a later stage of treatment without broadly affecting other OXPHOS complexes.

## 4 Discussion

This study investigated how semaglutide, tirzepatide and cagrilintide influence mitochondrial function in skeletal muscle under healthy and lipotoxic conditions. Interestingly, different patterns of mitochondrial adaptation were observed between the incretin‐based therapies. Semaglutide and cagrilintide both induced a short‐term reduction in mitochondrial respiration at 48 h that normalized with prolonged exposure. However, tirzepatide produced a delayed enhancement of mitochondrial capacity that was evident at 5 days, with no early suppression. Despite these functional differences, all three agents exhibited a consistent, transient modulation of Complex III at 48 h, changes that resolved by 5 days. The absence of alterations in mitochondrial DNA copy number indicates that these effects reflect reversible remodelling of the respiratory chain rather than altered mitochondrial biogenesis. Collectively, this pattern suggests that while incretin‐based therapies share a short‐term molecular signature at the level of OXPHOS regulation, their functional outcomes diverge, with tirzepatide demonstrating the most sustained enhancement of mitochondrial performance.

Under lipotoxic conditions, these compound‐specific responses were maintained, but became more pronounced. All three agents produced some degree of additional respiratory suppression at 48 h, reflecting the combined metabolic stress of palmitate exposure and drug treatment. However, only tirzepatide elicited a clear recovery and subsequent enhancement of mitochondrial function with prolonged exposure, restoring ATP production and maximal respiration and improving coupling efficiency by Day 5. In contrast, semaglutide and cagrilintide showed early impairments that normalized over time without functional improvement. These findings indicate that, although all three incretin‐based therapies create similar early mitochondrial responses, tirzepatide uniquely supports adaptive recovery under metabolic stress, suggesting an enhanced capacity to preserve or restore mitochondrial efficiency in lipid overloaded skeletal muscle.

A positive effect of GLP‐1 agents upon skeletal muscle mitochondrial function has been reported before both in vitro and in vivo. Intramuscular overexpression of GLP‐1 in mice resulted in improved exercise endurance, increased mitochondrial content and oxidative phosphorylation [18]. Furthermore, in C2C12 cells, exendin‐4 increased mtDNA copy number, basal and maximal respiration, proton leak and spare respiratory capacity [18]. These results align with our functional data presented here to indicate that incretin‐based therapies can act directly on skeletal muscle to positively modulate mitochondrial function. In contrast to this, our recent systematic review identified heterogenous and context‐dependent effects of GLP‐1RAs on skeletal muscle mitochondria [12]. The results of the review found evidence to support improvements in morphology and content, but inconsistent changes in respiratory function. These differences are most likely the result of differences in experimental design, metabolic content, duration of exposure and the GLP‐1RA used.

Of interest here is the divergent effect of the three GLP‐1 based therapies on mitochondrial function. The recovery of mitochondrial function observed with tirzepatide may reflect its dual activation of the GLP‐1 and the glucose‐dependent insulinotrophic polypeptide (GIP), thereby engaging a broader range of metabolic signalling than either the selective GLP‐1RA semaglutide or the amylin analogue cagrilintide. GLP‐1R activation has been shown to regulate mitochondrial content and function in skeletal muscle via AMPK‐dependent pathways [19]. Meanwhile, GIPR signalling has been implicated in the regulation of lipoprotein lipase (LPL) expression, triglyceride clearance and fatty acid uptake in oxidative tissues [20]. The dual activation achieved with tirzepatide may therefore enhance substrate flexibility and promote a more robust mitochondrial adaptation under metabolic stress. In contrast, semaglutide and cagrilintide, by acting on narrower signalling axes, may induce only a transient mitochondrial adjustment and lack the sustained oxidative enhancement seen with tirzepatide.

### 4.1 Limitations

A key limitation of this study is the use of C2C12 myotubes as an in vitro model, although the patterns seen in mitochondrial respiration were largely replicated in primary human myotubes. This cross‐species consistency strengthens the translational relevance of these findings. While these cells provide a controlled environment to investigate mitochondrial function and drug effect, they may not fully recapitulate the complexity of skeletal muscle physiology in vivo. This study looked at the amylin analogue as a single agent rather than in combination with a GLP‐1RA. Given the increasing clinical interest in dual GLP‐1/amylin agonism, further studies are warranted to assess the potential additive or synergistic effects of combined treatment on skeletal muscle mitochondrial function. Caution should be used when extrapolating these findings to clinical settings, with further studies using human in vivo models warranted to validate and extend on these results.

Future research should focus on elucidating the molecular signalling pathways that mediate the observed drug‐induced changes in mitochondrial function, with particular attention to key regulators of energy metabolism such as AMPK, SIRT1 and PGC‐1a. Understanding how these pathways are modulated could provide valuable insight into the mechanisms underlying the selective effects on mitochondrial efficiency and respiratory complexes. Additionally, it will be important to extend these findings by assessing functional outcomes relevant to muscle health, such as contractile performance and insulin sensitivity, to determine the physiological significance of mitochondrial modulation. Such studies will help bridge the gap between cellular bioenergetics and whole‐muscle function, guiding the development of more effective metabolic therapies.

In conclusion, this study demonstrates that incretin‐based therapies exert compound‐specific, time‐dependent effects on skeletal muscle mitochondrial function. Although semaglutide and cagrilintide induce transient modulation of respiration and OXPHOS expression, tirzepatide enhances mitochondrial function with prolonged exposure, particularly under lipotoxic conditions. These findings highlight both shared and divergent mechanisms among GLP‐1‐based therapies and suggest that dual receptor agonism may confer additional metabolic resilience through improved mitochondrial adaptation.

## Conflicts of Interest

Professor Melanie Davies has acted as a consultant/advisor and speaker for Eli Lilly, Novo Nordisk and Sanofi, has attended advisory boards for AbbVie, Amgen, AstraZeneca, Biomea Fusion, Carmot/Roche, Sanofi, Zealand Pharma, Regeneron, GSK and EktaH, and as a speaker for AstraZeneca and Boehringer Ingelheim. She has received grants from AstraZeneca, Boehringer Ingelheim and Novo Nordisk. Professor Pratik Choudhary reports personal fees from Novo Nordisk, Lilly and Sanofi. For the purpose of open access, the author has applied a Creative Commons Attribution licence (CC BY) to any Author Accepted Manuscript version arising from this submission.

## Supporting information


**Figure S1:** cell viability assay on C2C12 myotubes using increasing doses of Semaglutide for 48 h. Data are expressed as mean + SD (*n* = 3 biological replicates), statistical significance calculated between the following groups using Mann–Whitney *U* test: * for *p* < 0.05 for control vs. 400, 1 vs. 400, 10 vs. 400 nM; ** for *p* < 0.001 for control vs. 100 nM, control vs. 200 nM. Relative fluorescence units (RFU).
**Figure S2:** 48‐h cell viability assay on C2C12 myotubes using increasing doses of tirzepatide. Data are expressed as the mean ± SD (*n* = 3, biological replicates). Statistical significance calculated between the following groups using Mann–Whitney *U* test: * for *p* < 0.05 for control vs. 1 nM, control vs. 10 nM, control v2 100 nM, control vs. 200 nM and control vs. 400 nM; ** for *p* < 0.001 for 10 nM vs. 400 nM. Abbreviations Relative fluorescence units (RFU).
**Figure S3:** 48 h cell viability on C2C12 myotubes using increasing doses of cagrilintide. Data are expressed as the mean ± SD (*n* = 3, biological replicates), no statistical significance identified *p* = <0.3. Abbreviations: Relative fluorescence units (RFU).
**Figure S4:** (a) Representative microscope images of Oil Red O stained C2C12 myotubes after 12‐ and 24‐h exposure to different doses of palmitic acid, (b) percentage of lipids formed (%) after 24 and 48 h of palmitic acid doses.
**Figure S5:** Viability assay of C2C12 cells with doses of palmitic acid washed off after 24 h and left in SF media for 5 days.
**Figure S6:** Western blot expression of GLP‐1RA in human skeletal muscle.
**Figure S7:** Changes in mtDNA copy number in response to semaglutide, trizepatide and cagrilintide in healthy and PA‐treated C2C12 cells. (a) healthy C2C12's dosed with semaglutide, tirzepatide and cagrilintide for 48 h and 5 days, (b) PA‐conditioned C2C12's dosed with semaglutide, tirzepatide and cagrilintide for 48 h and 5 days. Data are presented as means ± SD (*n* = 6 per group). Statistical analysis was performed using one‐way ANOVA with Tukey's post hoc test or Kruskal–Wallis test with Dunn's post hoc test for nonparametric data.
