# Supplementary material for: Mitochondrial Adaptations in Skeletal Muscle Following Incretin‐Based Therapies: In Vitro
Source: J Cachexia Sarcopenia Muscle. 2026 Mar 19;17(2):e70254. doi: 10.1002/jcsm.70254 (PMC13140976; doi:10.1002/jcsm.70254)
Supplement: Supplementary file 1 — Figure S1: cell viability assay on C2C12 myotubes using increasing doses of Semaglutide for 48 h. Data are expressed as mean + SD (n = 3 biological replicates), statistical significance calculated between the following groups using Mann–Whitney U test: * for p < 0.05 for control vs. 400, 1 vs. 400, 10 vs. 400 nM; ** for p < 0.001 for control vs. 100 nM, control vs. 200 nM. Relative fluorescence units (RFU). Figure S2: 48‐h cell viability assay on C2C12 myotubes using increasing doses of tirzepatide. Data are expressed as the mean ± SD (n = 3, biological replicates). Statistical significance calculated between the following groups using Mann–Whitney U test: * for p < 0.05 for control vs. 1 nM, control vs. 10 nM, control v2 100 nM, control vs. 200 nM and control vs. 400 nM; ** for p < 0.001 for 10 nM vs. 400 nM. Abbreviations Relative fluorescence units (RFU). Figure S3: 48 h cell viability on C2C12 myotubes using increasing doses of cagrilintide. Data are expressed as the mean ± SD (n = 3, biological replicates), no statistical significance identified p = <0.3. Abbreviations: Relative fluorescence units (RFU). Figure S4: (a) Representative microscope images of Oil Red O stained C2C12 myotubes after 12‐ and 24‐h exposure to different doses of palmitic acid, (b) percentage of lipids formed (%) after 24 and 48 h of palmitic acid doses. Figure S5: Viability assay of C2C12 cells with doses of palmitic acid washed off after 24 h and left in SF media for 5 days. Figure S6: Western blot expression of GLP‐1RA in human skeletal muscle. Figure S7: Changes in mtDNA copy number in response to semaglutide, trizepatide and cagrilintide in healthy and PA‐treated C2C12 cells. (a) healthy C2C12's dosed with semaglutide, tirzepatide and cagrilintide for 48 h and 5 days, (b) PA‐conditioned C2C12's dosed with semaglutide, tirzepatide and cagrilintide for 48 h and 5 days. Data are presented as means ± SD (n = 6 per group). Statistical analysis was performed using one‐way ANOVA with Tukey' [file JCSM-17-e70254-s001.docx]

**Appendix 1. Viability test for drug dosing.**

**Methods:**

**Viability assay**

C2C12 cells were grown as previously described until confluent myotubes formed. Semaglutide (Bioserv, UK), tirzepatide (LKT Laboratories, USA), and cagrilintide (MedChem Express, USA) were initially diluted in SF media to a concentration of 1:1000 and further diluted into the SF media at concentrations of 1nM, 10nM, 100nM, 200nM and 400nM alongside a control with just SF media. Cells were incubated with the drug dose or control for 48hrs in triplicates. At the end of 48hrs, the Alamar Blue assay (Thermo Fisher Scientific, UK) was used the check cell viability. The reagent was diluted in serum free DF at a ratio of 1:10. This was added to each cell culture well and incubated for 3hrs. Post incubation, 100ul of the 1:10 solution from each cell culture well was added to a 96 well plate and read at 570nm on the MultiSkan. The cells were then lysed with radioimmunoprecipitation assay buffer (RIPA buffer) for protein analysis using the Biorad protein assay for normalization. These were frozen and stored until later analysis.

**Results:**


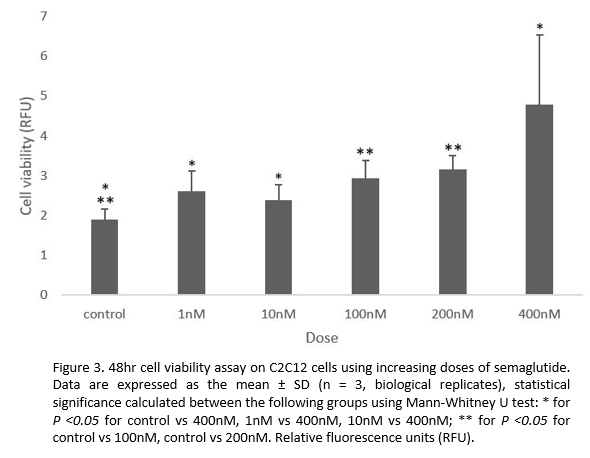


Figure 1. cell viability assay on C2C12 myotubes using increasing doses of Semaglutide for 48hr. Data are expressed as mean +- SD (n = 3 biological replicates), statistical significance calculated between the following groups using Mann-Whitney U test: * for P<0.05 for control vs 400nM, 1nM vs 400nM, 10nM vs 400nM; ** for P<0.001 for control vs 100nM, control vs 200nM. Relative fluorescence units (RFU).

The 48hr dose viability assay results are displayed in Figure 1. The non-parametric Krushal-Wallis H test was conducted after the Sharpiro-Wilk test showed the data as not normally distributed. Overall data showed statistical significance following the Mann-Whitney U test for the following group comparisons: *P <0.05* control = 1.9 ±- 0.26RFU vs 400nM = 4.79 ± 1.74RFU, 1nM = 2.61 ± 0.51RFU vs 400nM, 10nM = 2.38 ± 0.38RFU vs 400nM, control vs 100nM = 2.93 ± 0.46RFU, control vs 200nM = 3.15 ± 0.35RFU.


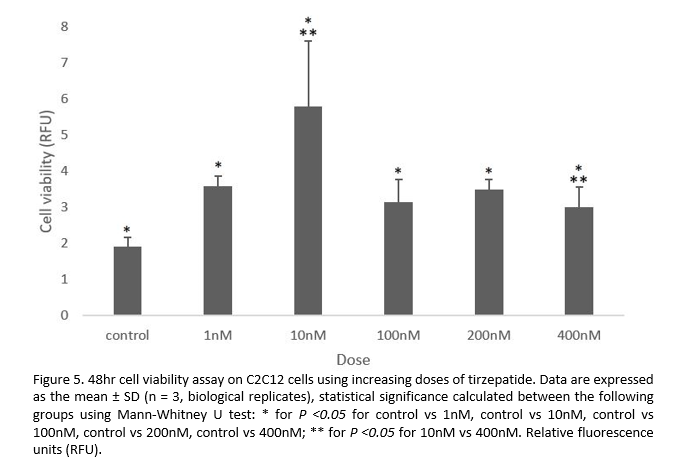


Figure 2. 48hr cell viability assay on C2C12 myotubes using increasing doses of tirzepatide. Data is expressed as the mean ± SD (n = 3, biological replicates). Statistical significance calculated between the following groups using Mann-Whitney U test: * for P <0.05 for control vs 1nM, control vs 10nM, control v2 100nM, control vs 200nM and control vs 400nM; ** for P <0.001 for 10nM vs 400nM. Abbreviations Relative fluorescence units (RFU).

The 48hr dose viability assay results for tirzepatide are displayed in Figure 2. Overall data showed statistical significance following the Mann-Whitney U test for the following group comparisons: *P <0.05* for control = 1.90 ± 0.26RFU vs 1nM = 3.59 ± 0.62RFU, control vs 10nM = 5.80 ± 1.81RFU, control vs 100nM = 3.15 ± 0.61RFU, control vs 200nM = 3.48 ± 0.28RFU, control vs 400nM = 3.00 ± 0.55RFU and 10nM vs 400nM.


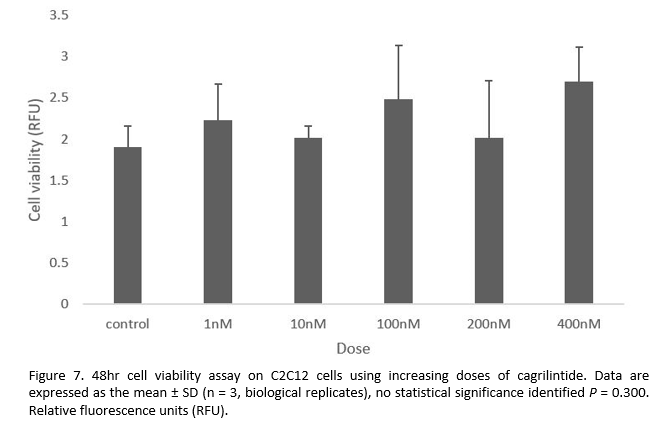


Figure 3. 48hr cell viability on C2C12 myotubes using increasing doses of cagrilintide. Data are expressed as the mean ± SD (n = 3, biological replicates), no statistical significance identified P=<0.3. Abbreviations: Relative fluorescence units (RFU).

The 48hr dose viability assay results for cagrilintide are displayed in Figure 3. Data showed: control = 1.90 ± 0.26RFU, 1nM = 2.23 ± 0.44RFU, 10nM = 2.02 ± 0.14RFU, 100nM = 2.48 ± 0.65RFU, 200nM = 2.02 ± 0.69RFU, 400nM = 2.7 ± 0.51RFU. The Shapiro-wilk test showed the data as not normally distributed so the non-parametric Krushal-Wallis H test was conducted which showed no statistical significance between groups (*P* = 0.3).

### Appendix 2. Viability test for palmitic acid dosing.

**Methods:**

**Oil Red O stain:**

To mimic the obese condition within the cells, palmitic acid (PA) was used. PA is a long-chain saturated fatty acid which can model obesity-related lipotoxicity, metabolic stress and insulin resistance. An Oil Red O stain was also completed to quantify lipid accumulation of the range of PA doses (0.0mM-1.0mM) at two time points (12hrs and 24hrs). PA doses were introduced to C2C12 myotubes (3 wells for each dose) 12hrs and 24hrs on chamber slides in SF media. At the two time points, the PA/SF media was rinsed off with PBS and the wells fixed with 4% paraformaldyhyde for 20mins. The slides were washed with 60% isopropanol for 5 mins and left to dry completely. Once dry the slides were submerged in oil red O solution for 30 minutes before being rinsed with 4 washes of ddH2O and 1 wash of 60% isopropanol. The slides were left to dry completely before imaging (15 images were taken of each dose). Measurements of lipid accumulation were taken using ImageJ by quantifying the amount of red stain in each image.


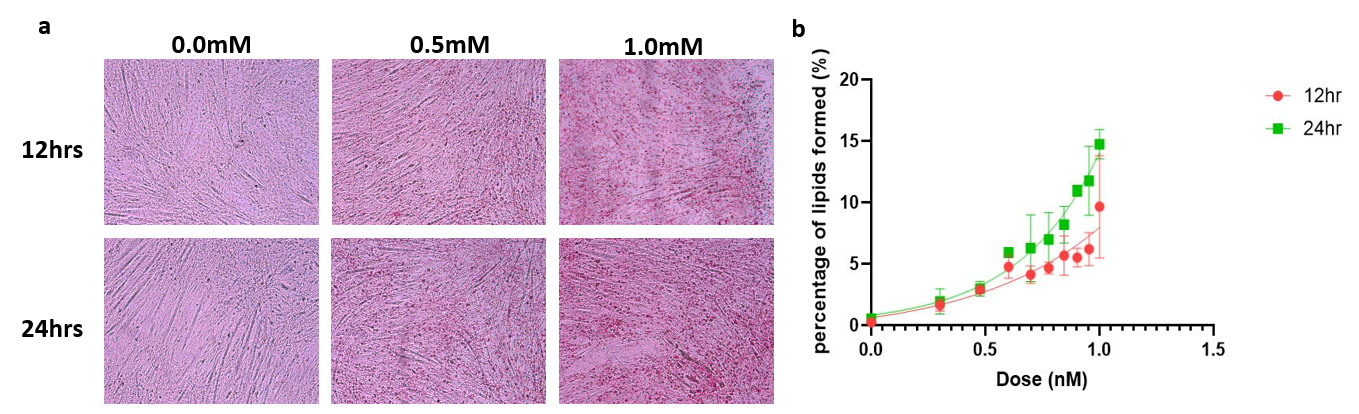


Figure 4. a) Representative microscope images of Oil Red O stained C2C12 myotubes after 12hrs and 24hrs exposure to different doses of palmitic acid, b) percentage of lipids formed (%) after 24hr and 48hr of palmitic acid doses.

**Viability assay:**

To ensure the optimal dosage used on the C2C12 myotubes does not negatively affect the cells after 5-day duration, the Alamar blue viability assay (Thermo Fisher Scientific, UK) was conducted on the same range of PA doses (0.0mM-1.0mM). Mature myotubes were dosed with PA (0.0mM-1.0mM) for 24hrs, rinsed with PBS and left in serum free media for 5 days. After 5 days, Alamar blue was diluted into SF media (1:10) in the wells and incubated for 3hrs. After 3hrs, 3 readings of each well (alamar blue/sf media) was taken on a spectrometer, read at 570nM.

**Results:**


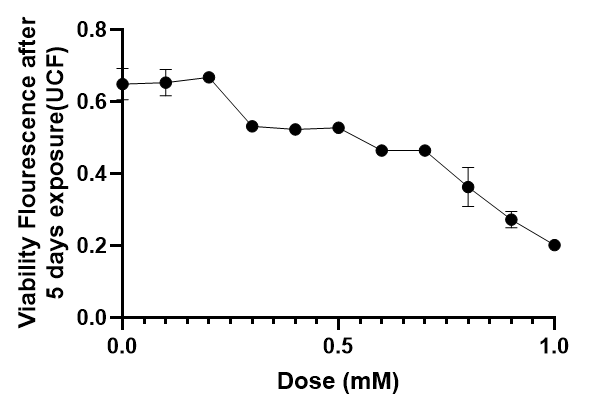
The viability assay showed that any doses higher than 0.2mM started to negatively affect the cells (Figure 5). As 0.2mM was the highest dose tolerable to the cells, this was the dose chosen moving forward to mimic the obesity state. Results of the lipid accumulation can be seen in (Figure 4).

Figure 5. Viability assay of C2C12 cells with doses of palmitic acid washed off after 24hrs and left in SF media for 5 days.


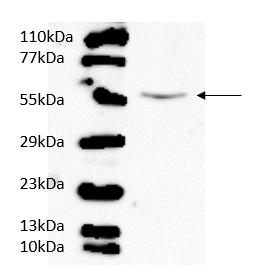
Appendix 3. GLP-1RA expression in skeletal muscle (Figure 6).

Figure 6. Western blot expression of GLP-1RA in human skeletal muscle.


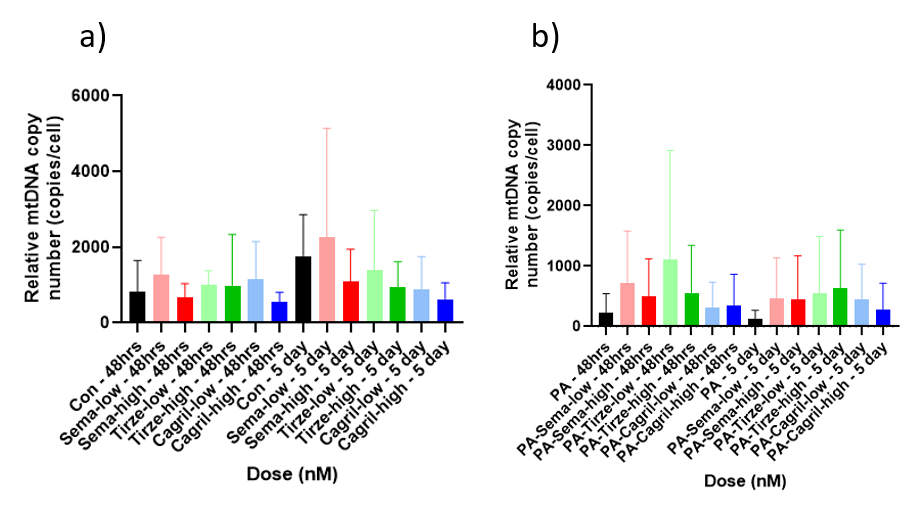
Appendix 4. Relative mtDNA copy number in response to semaglutide, tirzepatide and cagrilintide (figure 7).

Figure 7. Changes in mtDNA copy number in response to semaglutide, trizepatide and cagrilintide in healthy and PA-treated C2C12 cells. (a) healthy C2C12’s dosed with semaglutide, tirzepatide and cagrilintide for 48hrs and 5 days, (b) PA-conditioned C2C12’s dosed with semaglutide, tirzepatide and cagrilintide for 48hrs and 5 days. Data are presented as means ± SD (n = 6 per group). Statistical analysis was performed using one-way ANOVA with Tukey’s post hoc test or Kruskal-Wallis test with Dunn’s post hoc test for non-parametric data.
